# MUC1 and MUC5AC implication in Tunisian colorectal cancer patients

**DOI:** 10.3906/sag-2003-29

**Published:** 2021-02-26

**Authors:** Meriam HAZGUI, Marwa WESLATI, Rahma BOUGHRIBA, Donia OUNISSI, Dhouha BACHA, Saadia BOURAOUI

**Affiliations:** 1 Laboratory of Colorectal Cancer Research UR12SP14, Mongi Slim Hospital, La Marsa Tunisia; 2 Faculty of Sciences of Tunis, University of Tunis El Manar, Tunis Tunisia; 3 Department of Pathology and Cytology, Mongi Slim Hospital, La Marsa Tunisia

**Keywords:** Colorectal cancer, MUC1, MUC5AC, immunohistochemistry, RT-PCR

## Abstract

**Background/aim:**

Mucins, such as *MUC1* and *MUC5AC* , are known for their protective and moisturizing role in intestinal epithelium. Their expression is tightly controlled given their essential role in normal tissue homeostasis, whereas their deregulation leads to chronic inflammation, and even cancer. This study aimed to assess the expression profiles of *MUC1* and *MUC5AC* and their implications in colorectal carcinogenesis.

**Materials and methods:**

A retrospective study of 202 patients who underwent colorectal cancer (CRC) surgery was conducted. The expression of *MUC1* and *MUC5AC* was investigated by immunohistochemistry and reverse-transcription polymerase chain reaction (RT-PCR). Statistical analysis of mucin expression pattern, as well as the clinicopathological criteria of the patients, was performed using the chi-square test, survival curves were plotted using the Kaplan—Meier product-limit method, and differences between the survival curves were tested using the log-rank test.

**Results:**

The expression of both mucins was abnormally high in the tumor tissues for both mRNA and protein. *MUC1* expression was correlated with advanced cancer stages and lymph node metastases for both the mRNA (P < 0.016 and P < 0.002, respectively) and protein level (P < 0.006 and P < 0.001, respectively). However, *MUC5AC* expression did not pinpoint any significant association between the clinicopathological criteria, but patients who expressed *MUC5AC* showed an increase in overall survival (P < 0.009).

**Conclusion:**

The expression of *MUC1* might be a poor prognostic biomarker in CRC and could play a role in tumor transformation and metastasis. However, *MUC5AC* expression might be a good prognostic in the Tunisian cohort.

## 1. Introduction

The mucosal surface of the gastrointestinal tract is covered with mucus that is secreted by specialized epithelial cells, known as goblet cells. This mucus serves to lubricate, hydrate, and protect the epithelium against potential hazards [1,2].

As major components of mucus, mucins are high molecular weight glycoproteins, that heavily contain a large number of O-linked oligosaccharides and few N-glycan chains, linked to a protein backbone that is also known as mucin core protein or MUC. Over the years, many reports have highlighted the involvement of MUC expression in the invasion and metastasis formation of numerous malignancies, such as breast [3], gastric [4], and pancreatic carcinomas [5].

To date, a total of 22 mucins have been identified and classified into 2 subgroups: secreted and transmembrane [6]. Some of these mucins have been deemed as reliable prognostic biomarkers in many types of carcinoma prognosis. Nevertheless, findings are sometimes inconclusive, especially in colorectal cancer (CRC) [7,8]. Two mucin genes seem to be among the most studied in colorectal carcinoma: *MUC1* and *MUC5AC* [9–12].

The transmembrane mucin *MUC1* , located on chromosome 1q21-24, can be found on the apical surfaces of most epithelial cells, including those in the breast, digestive, respiratory, and genitourinary tract [6]. The secreted mucin *MUC5AC* , situated on chromosome 11p15.5, is usually expressed in the stomach and respiratory tract [13]. This mucin is normally absent in a healthy colon, but frequently present in colorectal adenomas and colon cancers [14]. Several researchers have reported an increase of both *MUC1* and *MUC5AC* expression when compared to the normal mucosa [2,15]. Consequently, *MUC1* could be a marker of poor prognosis, indicating that its upregulation may be involved in CRC progression [6,10]. In contrast, *MUC5AC* expression could be a good marker of better prognosis, and it increases the overall survival of patients [16]. 

Therefore, it was aimed to evaluate the expression pattern of both *MUC1* and *MUC5AC* in colorectal carcinoma, using immunohistochemical and molecular methods, to establish the possible links between their expressions and colorectal carcinoma prognosis.

## 2. Materials and methods

### 2.1. Patients and tumor samples

This retrospective study comprised 202 Tunisian patients who underwent CRC surgery between 2000 and 2017. The study protocol was approved by the Ethics Committee of Mongi Slim Hospital. The collected data included sex, age, tumor localization, histological type, differentiation, and tumor node metastasis (TNM) staging (stages I, II, III, and IV). TNM staging was based on the American Joint Committee on Cancer (AJCC) staging manual, eighth edition [17].

### 2.2. Immunohistochemical study

The 2-protein expression was analyzed on 4-µm thick formalin fixed paraffin-embedded samples. Slides were incubated in an oven at 37 °C overnight, deparaffinized in toluene, rehydrated in descending concentrations of ethanol, and finally in double distilled water. Later, they were immersed in citrate buffer (pH = 6), preheated in a microwave for 5 min to unmask the epitopes, and then kept at room temperature for 20 min, followed by a tris washing.

Peroxidase block was used to inhibit endogenous peroxidase activity. After washing 2 times in tris, *MUC1* (monoclonal mouse anti- *MUC1* protein antibody, catalog NCL- *MUC1* , clone Ma695, dilution 1:100; Novocastra Laboratories Ltd., Newcastle, UK) and *MUC5AC* (monoclonal mouse anti-Muc5AC protein antibody, catalog NCL- *MUC5AC* , clone CLH2, dilution 1:50; Novocastra Laboratories Ltd.) were incubated at room temperature for 1 h. After washing with tris, the sections were incubated with a postprimary block for 30 min. The revelation was conducted using 3,3’-diaminobenzidine (DAB) chromogen (Liquid DAB + substrate chromogen system; Novocastra Laboratories Ltd.), followed by nuclear staining using hematoxylin.

### 2.3. RNA extraction and reverse transcriptase polymerase chain reaction 

RNA extraction was performed on 34 frozen specimens taken from patients who underwent colorectal tumor resection at the Department of Surgery, and on 168 archival formalin-fixed, paraffin-embedded (FFPE) blocks, which were preserved at the Department of Pathology and Cytology of Mongi Slim Hospital, in La Marsa, Tunisia. 

With regards to the frozen specimens, the samples were kept at –80 °C in RNA later stabilization solution (Invitrogen, Thermo Fisher Scientific Inc., Carlsbad, CA, USA). First, the fragments were homogenized using a sterile, RNase-free mortar and pestle, and then the homogenates were immediately transferred into sterile, RNase-free microcentrifuge tubes that contained Trizol reagent (Invitrogen). The total RNA isolation was conducted as indicated in the manufacturer’s instructions, using the PureLink® RNA mini kit (Invitrogen).

For the FFPE samples, 3–8 pieces of 10-mm sections were placed into a sterile, RNase-free microcentrifuge tube and total RNA isolation was performed using the PureLink FFPE total RNA isolation kit (Invitrogen) according to the manufacturer’s instructions. Following RNA purification from both the frozen and FFPE specimens, a final DNase I digestion (Invitrogen) was performed to remove genomic DNA contamination. 

The RNA was stored at –80 °C until used. RNA concentration was determined using the Qubit RNA HS assay kit (Thermo Fisher Scientific, Waltham, MA, USA), and the quality was verified using an Agilent 2100 bioanalyzer (Agilent Technologies, Inc., Santa Clara, CA, USA), which analyzed 12 samples simultaneously, using Agilent RNA nano chips. The Agilent 2100 Expert software allows the automatic calculation of the RNA integrity number (RIN), which has a value between 1 and 10, where the higher this value is, the better the quality of RNA. Hence, using the RIN scores, samples with the highest value for both frozen and FFPE samples were chosen. 

The cDNA was synthesized using M-MLV reverse transcriptase (Invitrogen) in a 25 µL total volume reaction that contained 200 ng of total RNA for each sample.

The 35-cycle amplification included 94 °C for 30 s; 63 °C for 30 s for *MUC1* and 58 °C for 30 s for *MUC5AC* ; and 72 °C for 30 s. Glyceraldehyde-3-phosphate dehydrogenase (GAPDH) served as an internal standard. The polymerase chain reaction (PCR) primers for *MUC1* , *MUC5AC* , and GAPDH are detailed in Table 1. 

**Table 1:** Primers and cycling parameters.

Sequence (5’-3’)	Annealing temperature (°C)	Product length (bp)
MUC1F: CCAGCCCGGGATACCTACCATR: GCGACGTGCCCCTACAAGTT	63	188
MUC5ACF: CCTTCGACGGACAGAGCTACR: TCTCGGTGACAACACGAAAG	58	111
GAPDHF: GGGTGTGAACCATGAGAAGTR: GACTGTGGTCATGAGTCCT	57	136

The reverse-transcription (RT)-PCR products were verified on 2% agarose gel, and then stained with 0.1 mg/mL of ethidium bromide to check the amplification of the different genes under ultraviolet light.

After that, the *MUC1* and *MUC5AC* PCR products were analyzed using chip electrophoresis, and for that, the Agilent DNA 1000 chip kit was used. All of the chips were prepared according to the manufacturer’s instructions. It should be noted here that both the size and concentration of each separated band were automatically calculated using the software of the 2100 bioanalyzer.

### 2.4. Statistical analysis 

All of the clinicopathological criteria were analyzed using IBM SPSS Statistics for Windows 20.0 (IBM Corp., Armonk, NY, USA). Descriptive analysis and the categorical variables were evaluated using the chi-square test. 

For overall survival, the expression profiles of *MUC1* and *MUC5AC* were analyzed simultaneously, and 4 phenotypes were established, comprising *MUC* 1+ *MUC5AC* +, *MUC1* – *MUC5AC* –, *MUC1* + *MUC5AC* –, and *MUC1* – *MUC5AC* +, in addition to each gene separately.

Survival curves were plotted using the Kaplan–Meier product-limit method, and differences between the survival curves were tested using the log-rank test. P < 0.05 was accepted as statistically significant. 

## 3. Results

The 202 patients comprised 110 males (54%) and 92 females (46%), with a male to female sex ratio of 1.19. The average age was 60 years (ranging from 29 to 92 years). Tumors were more frequently located in the left colon (76%). Immunohistochemical study was performed on samples of paraffin-embedded tissues from 202 CRC patients. 

Determined were 54 cases of mucinous adenocarcinoma (27%), 28 cases of adenocarcinoma with mucinous component (mucin formation <50%) (14%), and 120 cases of nonmucinous carcinomas (59%). Normal colorectal mucosa from tissue margins served as the controls. The same samples were studied using RT-PCR, using both the frozen (34 cases) and paraffin-embedded tissues (168 cases).

Histologically, the series consisted of 164 well differentiated (81%), 30 moderately (15%), and 8 poorly differentiated (4%) samples. TNM staging system showed that 20 samples were stage I (10%), 80 were stage II (40%), 92 were stage III (45%), and 10 were stage IV (5%). 

### 3.1. Immunohistochemistry expression of MUC1

**Figure 1:**
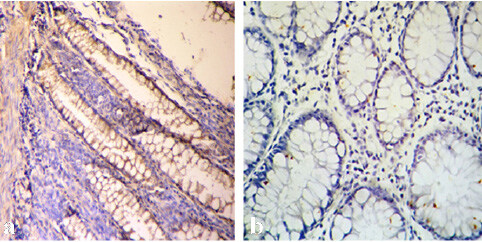
(a) Membranous and cytoplasmic positive staining with anti-MUC1 in healthy distant colorectal tissue (MUC1 ×200); (b) Absence of anti-MUC1 staining in healthy colorectal tissue (MUC1, ×400).

**Figure 2:**
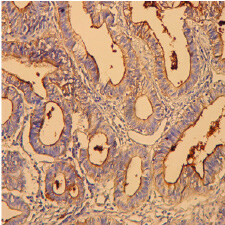
Positive staining with anti-MUC1 in the mucinous adenocarcinoma (MA) (MUC1 ×400).

Of the 202 samples of normal mucosa, 18 (9%) showed positive staining for *MUC1* in both the cytoplasm and apical membrane (Figure 1a), whereas the remaining samples reacted weakly or not at all (Figure 1b). *MUC1* expression was remarkably increased in the tumoral tissue when compared to the normal mucosa, where 88 of the 202 samples (44%) were positively stained in the apical membrane and cytoplasm (Figure 2).

Positive *MUC1* protein expression was determined in 54% of the stage III (50 of 92) and 60% of the stage IV (6 of 10) samples. In contrast, only 20% and 35%, respectively, of the stage I (4 of 20) and stage II (28 of 80) samples were positive. 

Only 30% of the patients with no lymph node metastases (N0) showed a positive signal for *MUC1* , whereas those with lymph nodes N1, N2, and N3 showed positive staining in 62%, 47% and 60% of the samples, respectively. Statistical analysis of the *MUC1* expression pattern along with the clinicopathological criteria of the patients showed that the signal positivity was strongly associated with the advanced cancer stages (P < 0.006) and lymph node metastasis (P < 0.001). 

The results were summarized in Table 2.

**Table 2:** MUC1 and MUC5AC protein expression profiles based on the clinicopathological criteria of patients.

Clinicopathological criteria	MUC1 immunostaining			MUC5AC immunostaining		
	Positiven = 88	Negativen = 114	P-value	Positiven = 30	Negativen = 182	P-value
Age (years)						
≥60 (n = 120)	46	74	0.070	16	104	0.463
<60 (n = 82)	42	40		14	68	
Sex						
Male (n = 110)	50	60	0.554	20	90	0.146
Female (n = 92)	38	54		10	82	
Tumor location						
Right colon (n = 48)	16	32	0.102	10	38	0.182
Left colon (n = 154)	72	82		20	134	
Histological type						
NMC (n = 120)	48	72	0.460	18	102	0.388
MA (n = 54)	26	28		10	44	
MC (n = 28)	14	14		2	26	
Differentiation						
Well (n = 164)	74	90		26	138	0.306
Moderate (n = 30)	12	18	0.487	2	28	
Poor (n = 8)	2	6		2	6	
Stages						
I (n = 20)	4	16	0.006	2	18	0.332
II (n = 80)	28	52		16	64	
III (n = 92)	50	42		10	82	
IV (n = 10)	6	4		2	8	
Lymph node metastasis						
N0 (n = 104)	32	72	0.001	18	86	0.332
N1 (n = 62)	38	24		8	54	
N2 (n = 34) °)	16	18		4	30	
N3 (n = 2)	2	0		0	2	

MA: mucinous adenocarcinoma, MC: mucinous component, NMC: nonmucinous carcinomas.

### 3.2. Immunohistochemistry expression of MUC5AC

**Figure 3:**
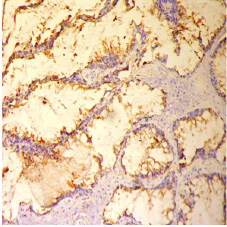
Positive staining with anti-MUC5AC in MA (MUC5AC ×200).

Since normal colorectal mucosa does not express *MUC5AC* , gastric mucosa was used as the positive control. The later showed a strong signal in the cytoplasm and the apical membrane. *MUC5AC* exhibited a similar staining pattern to that of *MUC1* in terms of the membrane and cytoplasm expression, which was observed in 30 of the 202 colorectal carcinomas samples (15%) (Figure 3). Statistical analysis of the *MUC5AC* immunohistochemical results compared with the different clinicopathological criteria did not show any significant association. The results are summarized in Table 2.

### 3.3. mRNA expression of MUC1 and MUC5AC in colorectal carcinomas

**Figure 4:**
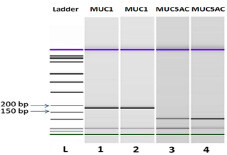
Bioanalyzer gel image of MUC1 and MUC5AC expression in the CRC tissues. Ladder at 1500 bp; MUC1 cDNA (188 bp); MUC5AC cDNA (111 bp).

*MUC1* gene expression was assessed using RT-PCR, revealing an amplicon of 188 bp in 48% of the samples (96/202). For the *MUC5AC* gene, amplification demonstrated that 40 of the 202 samples (20%) presented an amplicon of 111 bp. *GAPDH* , the reference gene, was successfully amplified in all of the samples (Figure 4).

A detailed analysis of all of the clinicopathological and immunohistochemical criteria compared with the gene molecular expressions was performed in order to identify any particularities (Table 3). *MUC1* was found to be highly involved in the advanced stages (P < 0.016) and lymph node metastasis (P < 0.002). Moreover, gene expression showed a significant correlation with the immunohistochemical results (P < 0.000). The *MUC5AC* statistical analysis highlighted only an association between the molecular expression and immunohistochemical profile (P < 0.000) (Table 3). 

**Table 3:** MUC1 and MUC5AC mRNA expression profiles based on the clinicopathological criteria of patients.

Clinicopathological criteria	MUC1 mRNA			MUC5AC mRNA		
	Positiven = 96	Negativen = 106	P-value	Positiven = 40	Negative	P-value
Age (years)					n = 172	
≥60 (n = 120)	53	67	0.248	22	98	0.526
<60 (n = 82)	43	39		18	64	
Sex						
Male (n = 110)	56	54	0.292	24	86	0.432
Female (n = 92)	40	52		16	76	
Tumor location						
Right colon (n = 48)	18	30	0.111	14	34	0.062
Left colon (n = 154)	78	76		26	128	
Histological type						
NMC (n = 120)	53	67	0.487	23	97	0.552
MA (n = 54)	29	25		13	41	
MC (n = 28)	14	14		4	24	
Differentiation						
Well (n = 164)	81	83		34	130	0.602
Moderate (n = 30)	13	17	0.356	4	26	
Poor (n = 8)	2	6		2	6	
Stages						
I (n = 20)	5	15	0.016	3	17	0.501
II (n = 80)	32	48		20	60	
III (n = 92)	53	39		15	77	
IV (n = 10)	6	4		2	8	
Lymph node metastasis						
N0 (n = 104)	37	67	0.002	23	81	0.516
N1 (n = 62)	39	23		13	49	
N2 (n = 34) °)	18	16		4	30	
N3 (n = 2)	2	0		0	2	
IHC profile			0.000			0.000
Positive	88	0		30	0	
Negative	8	106		10	162	

IHC: Immunohistochemistry.

### 3.4. Overall survival statistical analysis

Among the 202 patients, 24 were lost at follow-up; hence, these patients were not included in the survival curve analysis. The average overall survival rate of the 178 patients was 27 months (from 1 to 167 months). Among the 178 patients, 78 were *MUC1* positive (44%) and 26 were *MUC5AC* positive (15%). Moreover, 35% died during follow-up (62 patients), 16 were *MUC1* + *MUC5AC* +, 90 were *MUC1* – *MUC5AC* –, 62 were *MUC1* + *MUC5AC* –, and 10 were *MUC1* – *MUC5AC* +. The death rates were 62.5%, 27%, 35.5%, and 60%, respectively.

Based on the expression profiles of *MUC1* and *MUC5AC* simultaneously, the test did not pinpoint any significant association (P < 0.062). To further evaluate whether the expression status of *MUC1* or *MUC5AC* affected prognosis, each gene was studied separately.

**Figure 5:**
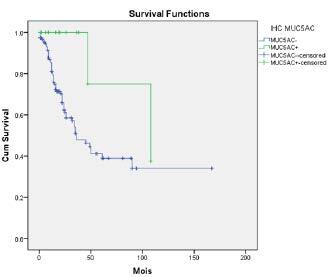
Patient survival curve.

Among the 62 deceased patients, 32 expressed *MUC1* (52%) and 16 expressed *MUC5AC* (26%). This percentage might suggest that *MUC5AC* expression could be a good prognostic biomarker, unlike *MUC1* , but statistically, an association was only found between *MUC5AC* expression and overall survival (P < 0.009) (Figure 5). Patients who expressed *MUC5AC* showed an increase in overall survival when compared to those who did not.

## 4. Discussion

As the third most common cancer, CRC has been tagged a preeminent public health concern and the main cause for the exponential morbidity rate throughout the world [18,19]. Mucins are large O-glycoproteins that are expressed on epithelia, which provide a protective barrier against mechanical, chemical, enzymatic, and microbial damage in the aerodigestive and genitourinary systems [20,21]. Mucin expression is tightly controlled, given their essential role in normal tissue homeostasis, whereas their deregulation leads to chronic inflammation, and even cancer. It has been accepted that quantitative and qualitative changes in mucins are not only a consequence, but also potential contributors to inflammation and cancer [15,22].

The current study focused on the evaluation of *MUC1* and *MUC5AC* implication in CRC, and the exploration of their possible use as markers in the detection and treatment of this neoplasm. The results highlighted that *MUC1* and *MUC5AC* mRNA, and protein levels, were notably upregulated in tumor tissues when compared with the normal mucosa. A similar pattern was also found in many other studies [2,10,15,23]. 

The immunohistochemical analysis showed the presence of *MUC1* in 9% of the studied normal mucosa. It should be noted that this percentage is variable, since some studies have reported a lack of *MUC1* expression in normal mucosa [10], while others have mentioned expression in some or all of the studied cases [15,24].

As for adenocarcinomas, many studies have reported a slight to moderate variation in the *MUC1* expression profile. In contrast to some other research, where the gene presence was noted in more than 80% of the cases, that herein was 44% [10,15,25]. This variability may have been attributed to either ethnicity and geographical distribution, or the heterogeneous designs of these studies (number of samples, sampling methods, tumor sites, tumor stages) [2].

In literature, the high expression of *MUC1* positively correlates with the stage, metastasis, poor tumor differentiation, and even worse, long-term survival [10,26–29]. The statistical analysis of the immunohistochemical profiles and mRNA expression of the patients herein showed that *MUC1* expression was relatively associated with advanced stages and lymph node metastasis [25,26]. Several studies have indicated *MUC1* involvement in colorectal carcinoma progression and metastasis development. One of the possible mechanisms is that this mucin acts as a ligand for cell adhesion molecules, which benefits the *MUC1* -expressing circulating tumor cells and helps them to adhere to endothelial cells, and therefore, allows their migration into secondary sites [2,27].

Under normal physiological conditions, secretory mucin *MUC5AC* is not expressed in the colonic mucosa, whereas its aberrant expression is observed during the development of colon cancer [1,13,15,23,30]. Since *MUC5AC* is not expressed in normal colon mucosa, gastric mucosa was used as the positive control. However, *MUC5AC* protein was found in 15% of the colorectal carcinomas (30/202), and similar results were published in other studies with varying percentages [10,13,15,21,31–33]. Although the current results regarding *MUC5AC* were in accordance with the aforementioned findings, its expression did not serve as a statistically significant marker of tumor progression. 

Regarding the mRNA expression, most of the samples with positive RT-PCR amplification showed positive immunohistochemical staining of the studied gene. Note that in some cases, the protein was not found, despite the presence of mRNA (8 samples with *MUC1* and 10 samples with *MUC5AC* ). The discordance between the transcript and protein expression levels probably would have been due to posttranscriptional mechanisms [34,35].

In the current study, it was not possible to find statistically significant relationships between the expression profiles of *MUC1* and *MUC5AC* simultaneously, and the survival of patients with CRC (P < 0.062). The analysis of each gene separately showed that patients who expressed *MUC5AC* had a better survival rate (P < 0.009). For *MUC1* , the evaluation was not significant (P < 0.889).

For *MUC5AC* , many studies have revealed that tumors with a negative expression of *MUC5AC* showed a worse prognosis when compared with tumors when *MUC5AC* was positive, while others studies have had the opposite result or did not pinpoint any correlations [1,9,30]. This supports the idea that the presence of *MUC5AC* can be a prognostic factor for nonaggressive colorectal carcinomas and a promising target in the treatment of colon cancer [23].

## 5. Conclusion

The expression of *MUC1* might be a biomarker for poor prognosis in CRC and could play a role in tumor transformation and metastasis. However, the expression of *MUC5AC* might have been a better prognostic in the Tunisian cohort. Given the importance of such analyses, future studies can be focused on the evaluation of other mucins, such as *MUC2* and *MUC4* , to demonstrate the involvement of their expression and establish a combination between them in colorectal carcinoma. Nonetheless, further investigation, in larger patient groups and with other techniques, is required to confirm the findings and predict the clinical usefulness of these molecular markers.
